# High-Performance Triboelectric Nanogenerators Based on Commercial Textiles: Electrospun Nylon 66 Nanofibers on Silk and PVDF on Polyester

**DOI:** 10.1021/acsami.2c13092

**Published:** 2022-09-23

**Authors:** Satyaranjan Bairagi, Gaurav Khandelwal, Xenofon Karagiorgis, Shravan Gokhool, Charchit Kumar, Guanbo Min, Daniel M. Mulvihill

**Affiliations:** † Materials and Manufacturing Research Group, James Watt School of Engineering, University of Glasgow, Glasgow G12 8QQ, U.K.; ‡ Bendable Electronics and Sensing Technologies (BEST) Group, James Watt School of Engineering, University of Glasgow, Glasgow G12 8QQ, U.K.

**Keywords:** textile triboelectric nanogenerator, wearable devices, electrospinning, silk and polyester, nylon 66, PVDF

## Abstract

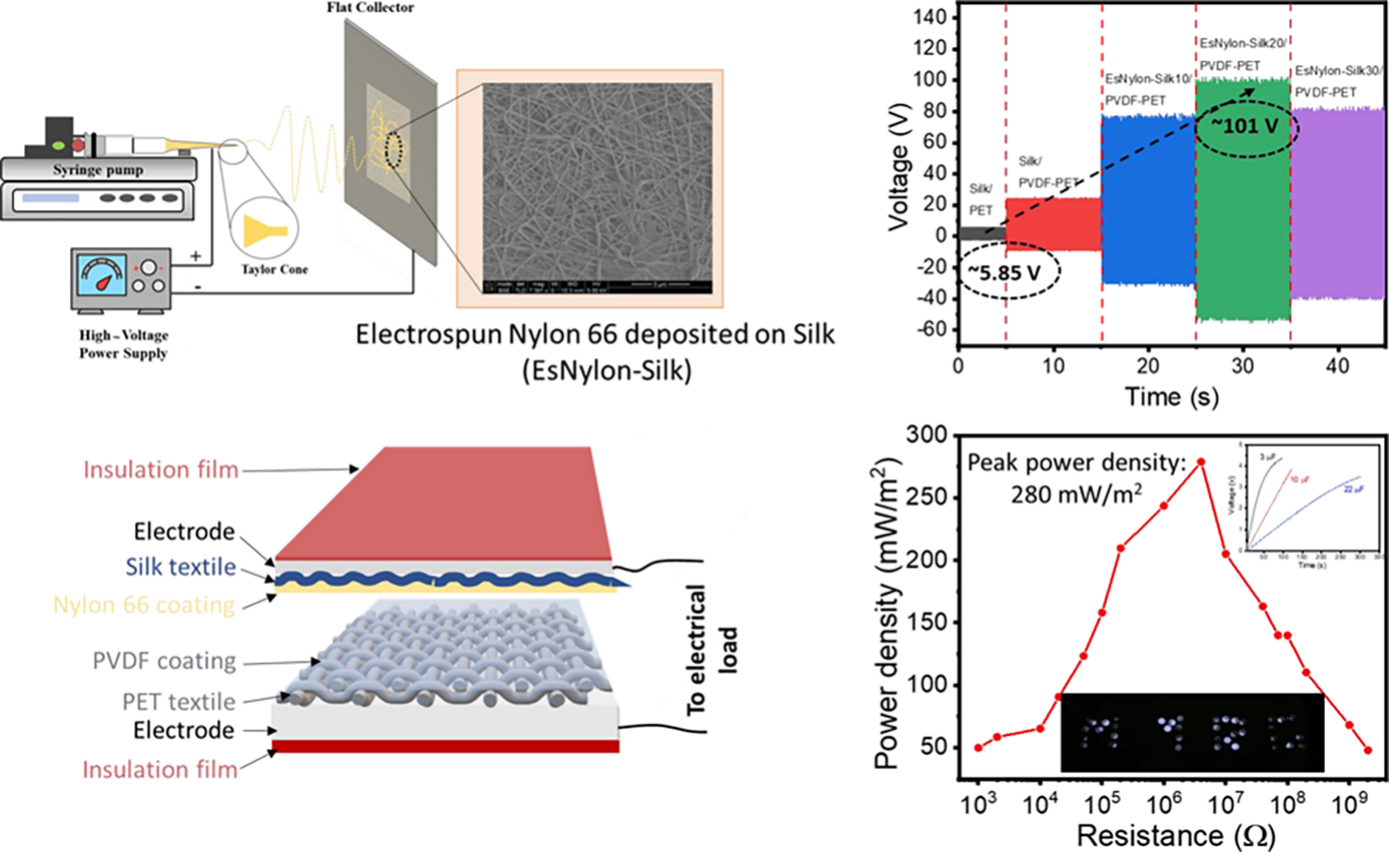

A high-performance textile triboelectric nanogenerator is developed based on the common commercial fabrics silk and polyester (PET). Electrospun nylon 66 nanofibers were used to boost the tribo-positive performance of silk, and a poly(vinylidene difluoride) (PVDF) coating was deployed to increase the tribo-negativity of PET. The modifications confer a very significant boost in performance: output voltage and short-circuit current density increased ∼17 times (5.85 to 100 V) and ∼16 times (1.6 to 24.5 mA/m^2^), respectively, compared with the Silk/PET baseline. The maximum power density was 280 mW/m^2^ at a 4 MΩ resistance. The performance boost likely results from enhancing the tribo-positivity (and tribo-negativity) of the contact layers and from increased contact area facilitated by the electrospun nanofibers. Excellent stability and durability were demonstrated: the nylon nanofibers and PVDF coating provide high output, while the silk and PET substrate fabrics confer strength and flexibility. Rapid capacitor charging rates of 0.045 V/s (2 μF), 0.031 V/s (10 μF), and 0.011 V/s (22 μF) were demonstrated. Advantages include high output, a fully textile structure with excellent flexibility, and construction based on cost-effective commercial fabrics. The device is ideal as a power source for wearable electronic devices, and the approach can easily be deployed for other textiles.

## 1 Introduction

The demand for wearable and portable electronic devices has increased tremendously in recent years. In addition, the rapid development of the Internet of Things concept has encouraged the wide application of various low-power-consumption electronic devices. Therefore, there is heightened focus on the provision of a sustainable power source for these devices.^1−5^ At present, wearable devices rely on battery packs to guarantee reliability of power supply, but these are cumbersome and also not environmentally friendly. Therefore, it is desirable if the wearable power problem can be solved using sustainable and renewable sources. In fact, freely available sources of mechanical energy are abundant in our world. Examples include wind, water wave, machine vibrations, and everyday human body motion. Much of this energy is actually wasted, and harvesting it is one of the most pressing challenges at present.^6^ In this regard, nanogenerators are good candidates since they have the capability to harvest electrical energy from different dispersed environmental energy sources. The various nanogenerators such as piezoelectric, thermoelectric, pyroelectric, and triboelectric nanogenerators have drawn great attention owing to their simple configuration, light weight, and cost-effective fabrication.^7−9^ Triboelectric nanogenerators are particularly promising for wearables due to their high efficiency at the low frequencies typical of human motion (∼2 Hz).^10−13^ They are also cost effective as their output depends on the repeated contact (or sliding) of rather simple materials, and their output is higher than for piezoelectric technology. However, conventional triboelectric nanogenerators based on solid films do have some limitations such as their lower flexibility, and this can limit their application in wearable applications where flexibility and breathability are paramount.^14^ In this context, textile-based triboelectric nanogenerators (t-TENGs) have gained significant attention.^15^ For example, Lee et al.^16^ developed a nanofiber-TENG based on a PVDF/cellulose nanocrystal textile fabric. The “as-fabricated” t-TENG was found to exhibit an output voltage, a current density, and a power density of ∼2 V, ∼1.55 mA/m^2^, and ∼2.19 mW/m^2^, respectively, at 9.8 kPa pressure. Similarly, in another study, a t-TENG was developed using polyester/AgNWs/GO/PMMA and polyester/AgNWs/GO/PDMS. Here, it was found that the developed t-TENG could generate an output voltage of ∼4 mV, an output current of ∼2 μA, and a power density of ∼0.07 mW/m^2^, respectively.^17^ However, the power output from t-TENGs has so far been significantly lower than for conventional film-based TENGs. Therefore, to enhance electrical performance, different processes such as chemical modification, plasma treatment, and structural optimization of the textiles have already been explored by different research groups.^18,19^ However, as we highlighted in Gokhool et al.,^20^ one of the major causes of the low output is likely to be the low contact area developed between the discrete fibers at a textile interface. Recent work on film-based TENGs has actually confirmed that the electrical output is highly sensitive to the amount of “real contact area” developed^21,22^ (for obvious reasons: electrons need contact in order to move across the interface). Therefore, a good approach to enhancing the t-TENG output would appear to be that of boosting the contact area developed at the textile interface. In this regard, deposition of electrospun nanofibers on the textile surface could be a good approach to enhance the contact area between the two layers. Nanofibrous layers have a higher surface to volume ratio and greater areal density of fibers, which will naturally promote more contact area in the t-TENG.^23−25^ A number of recent papers have deployed electrospun nanofibers in triboelectric nanogenerators.^26−32^ A comprehensive review is given in Babu et al.^33^ They found that nanofiber-based TENGs can demonstrate better electrical performance perhaps due to greater conformity with the countersurface and higher porosity.^34−38^ The higher porosity of the electrospun web means it contains a higher volume of air, thus conferring a higher dielectric constant. Greater conformity with the countersurface is likely to generate increased contact area. The higher dielectric constant and contact area can confer enhanced electrical performance for nanofiber-based TENGs.^23,39^ However, electrospun nanofibers do have lower mechanical properties as compared to microfibers.^40,41^ Therefore, we deposit the electrospun layer onto a conventional woven fabric to provide the required mechanical strength and stability. In this work, nylon 66 electrospun nanofibers are deposited on a silk woven fabric to form the tribo-positive layer. To the best of our knowledge, this is the first time that deposition of electrospun nanofibers on woven fabric has been used to construct a fully textile high-performance t-TENG, which is based on commercially available textiles, is suitable for wearable applications, and possesses competitive output performance.

Another very important aspect in enhancing the electrical performance of the t-TENG is the selection of a material pair with the widest possible separation on the triboelectric series.^42,43^ In the present work, silk and polyester (PET) woven fabrics have been chosen as the base fabrics. This is because silk is tribo-positive, PET is tribo-negative, and both are widely used commercial fabrics. To further enhance the electrical performance, the silk fabric is coated with electrospun nylon 66 and the PET with PVDF. The reasoning behind this (in addition to the advantages mentioned for nanofibers) is that nylon 66 is more tribo-positive than silk and PVDF is more tribo-negative than PET. After developing the triboelectric layers, different characterization techniques are utilized such as field emission scanning electron microscopy (FE-SEM) and 3D optical profilometry for surface morphology analysis as well as X-ray diffraction (XRD) and Fourier transform infrared (FTIR) for structural analysis. Finally, the electrical performance of the t-TENG is characterized using an electrodynamic shaker in normal contact separation mode. The optimized t-TENG device in the present work (EsNylon-Silk20/PVDF-PET) produced a maximum output voltage and a short-circuit current density of ∼101 V and ∼24.5 mA/m^2^, respectively, with a max power density of ∼280 mW/m^2^.

## 2 Fabrication and Testing

### 2.1 Materials

Conventional silk and PET plain woven fabrics (Dalston Mill Fabrics, London) were used as the substrates for nylon and PVDF layers. Nylon 66 and PVDF polymer chips as well as formic acid and dimethylformamide were procured from Sigma-Aldrich, UK. All chemicals were used without further purification.

### 2.2 Methods

#### 2.2.1 PVDF Coating on Polyester Woven Fabric

**Figure 1:**
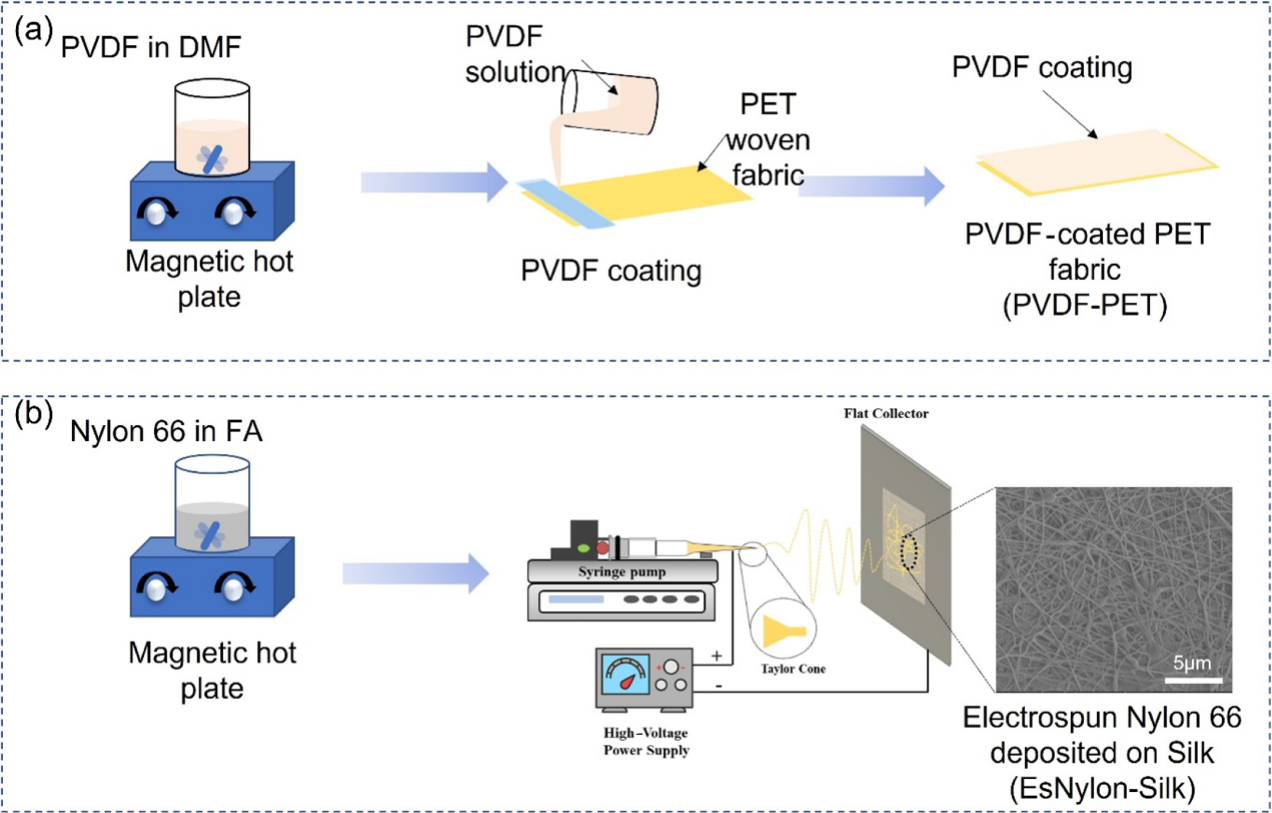
(a) Flow-chart outlining PVDF polymer coating on PET fabric (PVDF-PET) and (b) schematic diagram describing nylon 66 electrospun nanofiber deposition on silk fabric (EsNylon-Silk).

Here, PVDF polymer solution was coated on the PET woven fabric by hand. For this, 30% (w/v) PVDF polymer chips were dissolved in DMF solvent by continuous magnetic stirring at 80 °C until a clear transparent solution occurred. Thereafter, PVDF solution was coated on the PET fabric by hand using a glass bar followed by drying at 100 °C for 2 h (Figure 1a). The dried samples were then stored in a plastic box for further processing. The PVDF-coated PET is designated hereafter as PVDF-PET.

#### 2.2.2 Deposition of Nylon 66 Electrospun Nanofibers on Silk Woven Fabric

For deposition of the nylon 66 electrospun web, first, 20% (w/v) nylon 66 polymer was dissolved in formic acid by continuous magnetic stirring for 12 h at room temperature. The as-prepared nylon 66 solution was then poured into a 20 mL syringe for electrospinning (TL-PRO, TONGLI TL, China). A piece of silk woven fabric having dimensions of 10 × 10 cm^2^ was fixed on a flat collector maintaining a 10 cm distance between the needle tip and flat collector. During electrospinning, a 27 kV external voltage was applied into the nylon 66 polymer solution having a flow rate of 0.01 ml/h. Electrospinning fiber deposition times of 10, 20, and 30 min were used. The deposited electrospun-nylon (EsNylon) nanofiber matts (with silk substrates) generated at these deposition times are designated as EsNylon-Silk10, EsNylon-Silk20, and EsNylon-Silk30, respectively. After electrospun fiber deposition, all samples were cured for 3 h at 60 °C to remove residual solvent. A schematic of the electrospinning deposition of nylon 66 fibers is shown in Figure 1b.

### 2.3 t-TENG Fabrication, Characterization, and Testing

**Figure 2:**
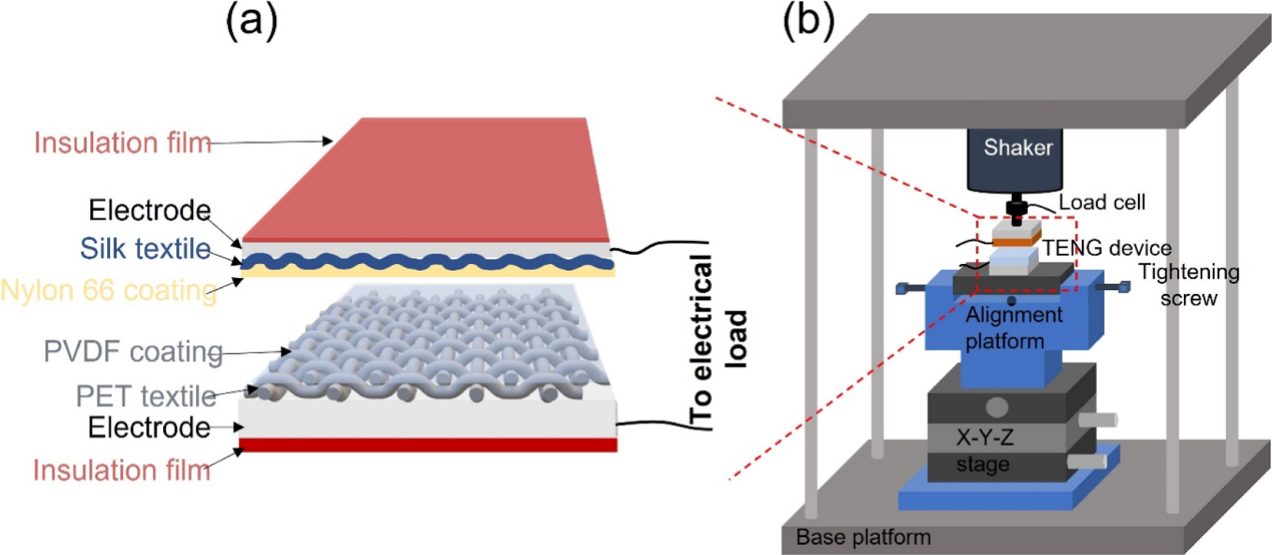
(a) Schematic representation of the t-TENG construction and (b) schematic diagram of the vertical contact separation mode test setup.

To fabricate the t-TENG, the woven silk fabric with deposited electrospun nanofibers (nylon 66) and PVDF-coated PET woven fabric were cut into pieces having dimensions of 2.5 cm × 2.5 cm (the final TENG size). Subsequently, conductive aluminium electrodes were pasted on one side of the triboelectric layers followed by insulation of the electrodes using Kapton tap. Conductive copper lead wires were connected on the aluminum electrode to capture signals from the triboelectric nanogenerator. The final make-up of the t-TENG is shown in Figure 2a.

The developed triboelectric layers (nylon 66 electrospun nanofibers deposited on silk woven fabric and PVDF-coated PET woven fabric) were then characterized by various techniques. A field-emission scanning electron microscope and a 3D optical profilometer (Alicona InfiniteFocus) were used to analyze the surface morphology of the developed triboelectric layers (Digital Surf MountainsMap software was utilised to create 3D surface images). FTIR spectroscopy (using a Bruker VERTEX 70 spectrometer) and XRD (using a PANalytical X’Pert Pro diffractometer) were used to study the structural properties of the triboelectric layers.

The electrical output of the t-TENG was then characterized using an electrodynamic shaker (TIRA, TV 50018, Germany) in vertical contact separation mode with a maximum separation of 1 mm. A schematic representation of the test setup is given in Figure 2b. The rig allows control over the frequency, contact force, and distance between triboelectric layers. The electrical performance of the t-TENG was measured with a digital oscilloscope (MSO-X 4154 A, Keysight, USA) including voltage and short-circuit current. The oscilloscope was connected with an operational amplifier and a voltage divider circuit (resistances of 1 KΩ and 2 G Ω) to ensure that the impedance of the voltage meter setup was much larger than the TENG internal impedance. During measurement of the short-circuit current, an equivalent circuit of a low noise current amplifier (Stanford Research, SR570) was utilized to accurately measure the t-TENG current (as TENGs produce currents in the microampere range). Tests were carried out on pristine silk against PET (Silk/PET), pristine silk against PVDF-coated PET (Silk/PVDF-PET), and electrospun nylon 66 nanofiber-coated silk in contact with PVDF-coated PET (EsNylon-Silk/PVDF-PET) for deposition times of 10, 20, and 30 min.

## 3 Results and Discussion

### 3.1 Morphological Analysis

**Figure 3:**
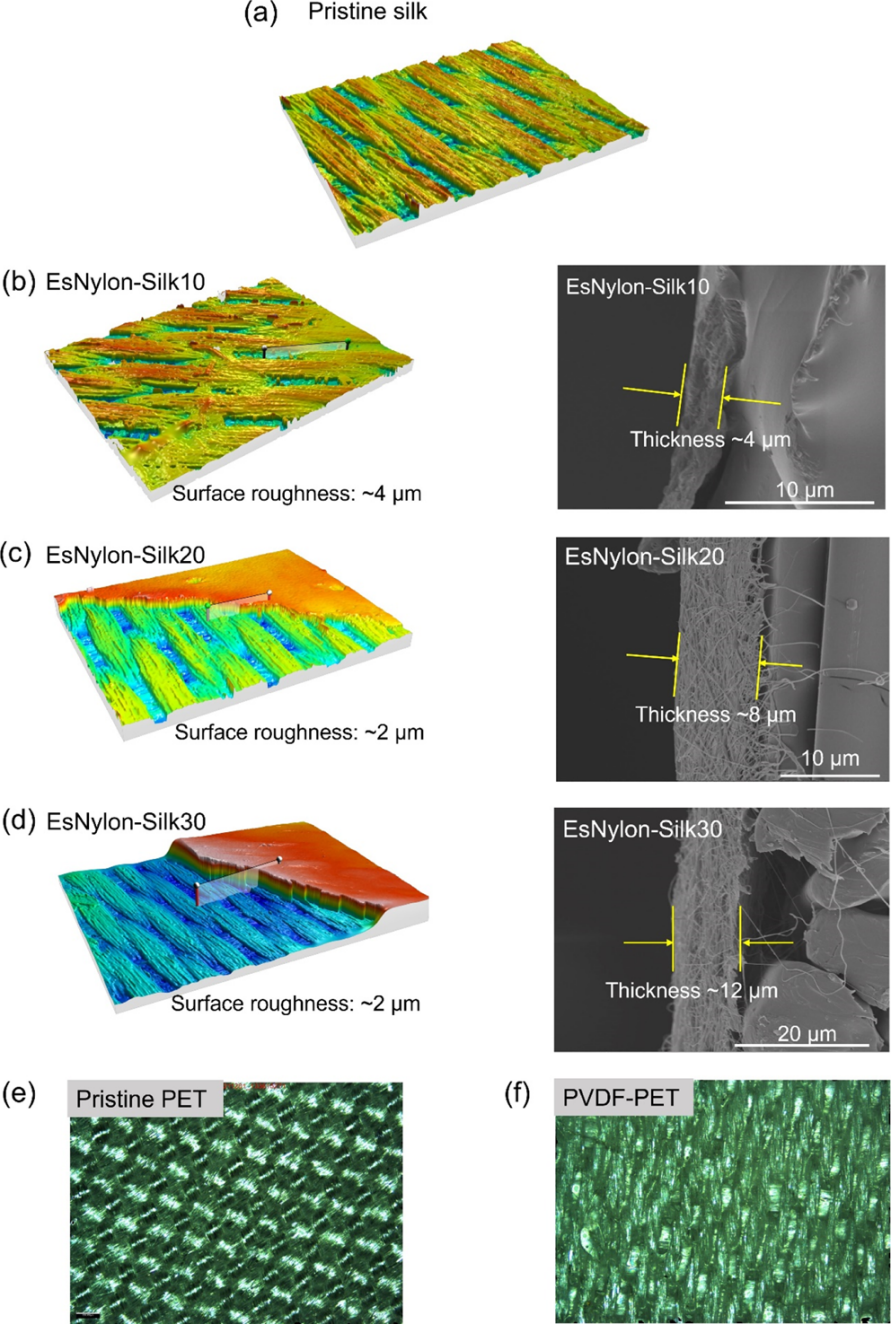
(a) Surface morphology of the raw silk woven fabric; (b–d) 3D surface scans (left) and cross-sectional SEM images (right) indicating progressively increasing thickness of the deposited nylon 66 nanofibrous layer on the silk woven fabric substrate with deposition times of (b) 10, (c) 20, and (d) 30 min; (e) surface morphology of the pristine PET woven fabric and (f) morphology of the PVDF-coated PET woven fabric.

Figure 3 shows the surface morphology of the fabrics including pristine silk woven fabric (Figure 3a), together with 3D surface scans (left) and cross-sectional SEM images (right) indicating the thickness of the nylon 66 electrospun nanofiber layer on the silk fabric (EsNylon-Silk) at deposition times of 10 min (Figure 3b), 20 min (Figure 3c), and 30 min (Figure 3d). The 3D scans were obtained via optical profilometry (Alicona InfiniteFocus). It can be seen from Figure 3b–d) that the thickness of the electrospun nanofiber layer progressively increases with electrospinning deposition time. Table 1 gives the layer thickness values together with values for root mean square (RMS) surface roughness. The thickness of the electrospun layer is ∼4 μm at 10 min of deposition, ∼8 μm for 20 min of deposition, and ∼12 μm for 30 min of deposition, as measured from the cross-sectional SEM images using Image J software (Figure 3b–d). In addition, the areal RMS surface roughnesses *S*q of the electrospun nanofiber webs were evaluated as ∼4 μm (EsNylon-Silk10), ∼2 μm (EsNylon-Silk20), and ∼2 μm (EsNylon-Silk30). This indicates that the roughness perhaps reaches a steady value after some critical level of deposition. Surface morphologies for the pristine PET woven fabric and PVDF-coated PET fabric are given in Figures 3e,f, respectively. The impact of the PDVF coating in the optical image in Figure 3f is clearly visible when compared to the image of the pristine PET in Figure 3e. For better understanding, the surface morphology of the PVDF-coated PET layer measured by FE-SEM is provided in Figure S1. The thickness of the PVDF coating on the PET woven fabric is around 40 μm when measured by a micrometer.

**Table 1:** Areal RMS Surface Roughness (*S*_q_) and Thickness (*d*) of the Nylon 66 Electrospun Nanofiber Web Deposited on the Silk Woven Fabric

Sl. no.	type of surface	coating thickness (*d*) (μm)	surface roughness (S_q_) (μm)
1.	silk		
2.	EsNylon-Silk10	∼4	4
3.	EsNylon-Silk20	∼8	2
4.	EsNylon-Silk30	∼12	2

### 3.2 Structural Analysis

**Figure 4:**
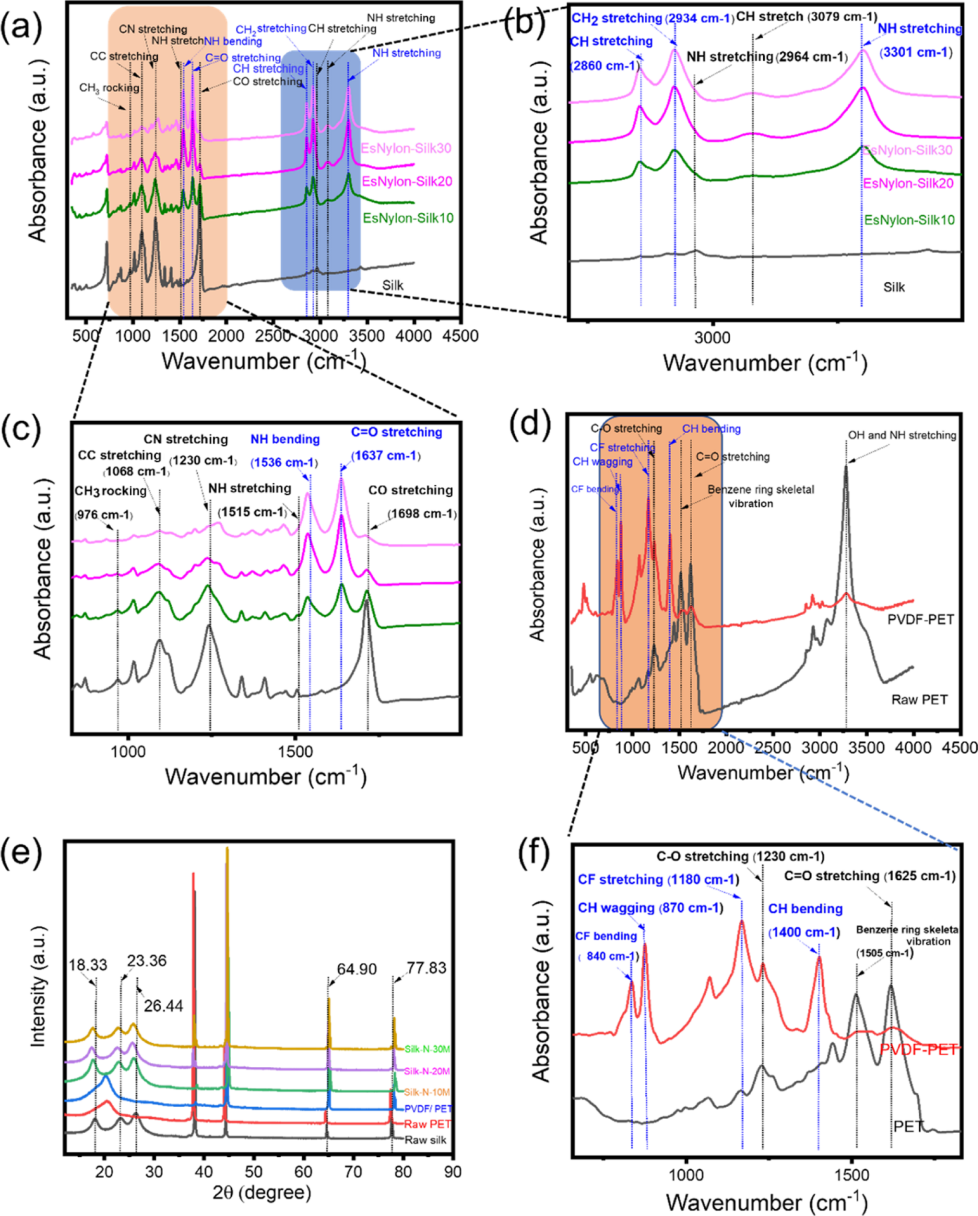
(a) IR spectra for raw silk woven fabric and electrospun nylon 66 nanofiber-coated silk fabrics at 10, 20, and 30 min deposition times; (b,c) magnified zoomed-in views corresponding to the shaded regions in (a); (d) IR spectra for raw PET woven fabric and PVDF-coated PET fabrics; (e) XRD patterns of the raw silk woven fabric, raw PET woven fabric and nylon 66-coated silk woven fabrics at 10, 20, and 30 min of deposition times and (f) magnified image of the selected shaded region in (d).

Figure 4a shows the FTIR analysis of the pristine silk woven fabric and the nylon 66 electrospun-coated silk woven fabric for different fiber deposition times. Two small peaks are visible at wavenumbers of 2964 and 3079 cm^–1^. These correspond to N–H stretching of the amide B and CH stretching of the peptide chain in the silk, respectively. Additionally, −CO stretching and N–H stretching vibration bands appear at 1698 and 1515 cm^–1^, respectively, in the IR spectrum of silk. Three additional peaks can also be observed at wavenumbers of 1230, 1068, and 976 cm^–1^ in the IR spectrum of the silk fabric: these peaks mainly correspond to CN stretching, CC stretching, and CH_3_ rocking.^44−46^ Similarly, it can be seen from IR spectrums of the nylon 66 electrospun nanofiber-coated silk fabrics that different peaks are also visible at wavenumbers corresponding to the presence of nylon 66. For example, the nylon 66 electrospun-coated silk fabric exhibited peaks at wavenumbers of 3301 and 2934 cm^–1^ corresponding to N–H stretching vibration and −CH_2_ stretching vibration, respectively, in nylon 66. Additionally, peaks exhibited at wavenumber 2860, 1637 and 1536 cm^–1^ correspond to the presence of −CH symmetric stretching vibration, −C=O stretching vibration, and N–H bending vibration in the nylon 66, respectively.^47,48^ These characteristic signatures on the IR spectrum confirm the successful deposition of nylon 66 on the silk fabrics. To give a clearer view, magnified views along with corresponding wave numbers are shown in Figure 4b,c. Figure 4d shows the FTIR spectra for the pristine PET woven fabric and the PVDF-coated PET woven fabric. A strong absorption peak at 3280 cm^–1^ can be assigned to the −OH and N–H groups in the PET fabric. In addition, three additional peaks at 1625, 1505, and 1230 cm^–1^ are characteristic of the presence of C=O stretching vibration, benzene ring skeletal vibration, and C–O stretching vibration in the PET fabric, respectively.^49−51^ On the other hand, the PVDF-coated PET fabric shows peaks at 1400, 1180, 870, and 840 cm^–1^, characteristic of C–H bending, C–F stretching, C–H wagging, and C–F bending in the PVDF polymer.^52−54^ The peak at 840 cm^–1^ implies a β crystalline phase of the PVDF polymer as shown in Figure 4f. Hence, the presence of the PVDF polymer on the surface of the PET fabric is confirmed by the FTIR results.

For further validation of the FTIR results, XRD analysis was carried out for the samples. Figure 4e shows XRD patterns of the different samples such as for raw silk, raw PET, PVDF-coated PET, and nylon 66 electrospun nanofiber-coated silk fabrics. Generally, silk fibroin has two forms: Silk I and Silk II. Silk I mainly exists in the raw silk gland before spinning, while silk II is present after spinning of Silk I. Therefore, silk fabric (as Silk II) should be the one used in the construction of the t-TENG. Indeed, the Silk II structure can be confirmed by peaks exhibited at 2θ equal to 18.33, 23.36, and 26.44° in the XRD pattern of the silk woven fabric.^55−57^ On the other hand, it can be seen from the XRD patterns for the nylon 66-coated silk fabric that all peaks corresponding to 2θ of 18.33, 23.36, and 26.44° are shifted toward lower angles compared to pristine silk woven fabric, thus confirming the deposition of the electrospun nylon 66 on the silk woven fabric.^58^

### 3.3 Triboelectric Properties

**Figure 5:**
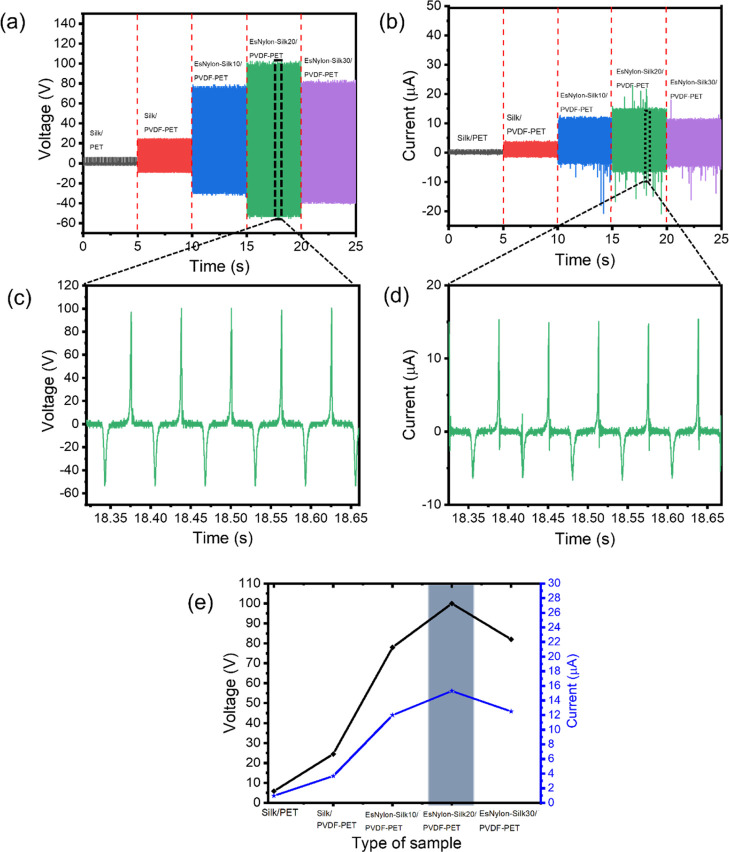
t-TENG electrical output. Results are shown for pristine silk against PET (Silk/PET), pristine silk against PVDF-coated PET (Silk/PVDF-PET), and electrospun nylon 66 nanofiber-coated silk in contact with PVDF-coated PET (EsNylon-Silk/PVDF-PET) for deposition times of 10, 20, and 30 min: (a) output voltage; (b) output current; (c) magnified voltage signal; (d) magnified current signal; and (e) voltage and current vs sample type. Contact force: 8 N (12.8 kPa), frequency 8 Hz.

Output voltage and current are shown in Figure 5 for pristine silk against PET (Silk/PET), pristine silk against PVDF-coated PET (Silk/PVDF-PET), and electrospun nylon 66 nanofiber-coated silk in contact with PVDF-coated PET (EsNylon-Silk/PVDF-PET) for deposition times of 10, 20, and 30 min. For these results, the contact force and frequency were held constant at 8 N (12.8 kPa pressure) and 8 Hz, respectively. Referring to Figure 5a, the output voltage is ∼5.85 V for the baseline Silk/PET pairing. This increases 4.2 times to ∼24.5 V when the PET is coated with the more tribo-negative PVDF. The addition of the electrospun nylon nanofibers on the silk then significantly boosts the output to ∼78 V (for 10 min of deposition time) and to a maximum of ∼100 V for 20 min of deposition time. The results for the output current are similar (Figure 5b). The output current is ∼0.97 μA for Silk/PET, ∼3.7 μA for Silk/PVDF-PET, ∼12 μA for EsNylon-Silk/PVDF-PET at 10 min of deposition time and a maximum of 15.3 μA for EsNylon-Silk/PVDF-PET at 20 min of deposition time. The EsNylon-Silk/PVDF-PET result at 20 min of deposition time represents an enormous ∼17 times’ increase in voltage and ∼16 times’ increase in current over the pristine Silk/PET[(see corresponding video in the Supporting Information (Video S1)]. Thus, the route explored here offers significant potential for boosting the performance of commercial silk and PET-based wearable TENG systems. The reasons for the large boost are likely to be a combination of the increased tribo-positivity of the nylon (plus tribo-negativity of the PVDF) and increased real contact area afforded by the electrospun nanofibers. Contact area likely increases because of a far greater areal fiber contact density and because the nanofibers are more likely to be able to conform to the counter-surface. The presence of amine groups in the nylon is likely to confer greater electro-positivity, and the concentration of amine groups would be expected to increase with deposition time. This helps explain the increase in output going from 10 to 20 min of deposition time. However, the output voltage and current actually drop off somewhat when the deposition time is increased further to 30 min (Figures 5a,b). Even though the amine group concentration is expected to be highest at 30 min of deposition time, we observed that the higher layer thickness at 30 min (∼12 μm—see Section 2.2.1) resulted in a tendency for the electrospun layer to peel off from the fabric substrate and thus reduce the TENGs’ ability to induce charges on the electrodes. This indicates an important practical limitation in selecting the optimum deposition time unless adhesion with the substrate can be improved. There is a second reason why high thickness might reduce output in the 30 min case: the distance-dependent electric field model based on the Maxwell equation indicates that TENG output decays with increasing thickness between the tribo-contact surface and electrode.^59^ This is because the electric field generated by the charged surfaces decays with distance, which results in less induced charges if the electrode is further away. Magnified views of the voltage and current signals for EsNylon-Silk20/PVDF-PET are shown in Figure 5c,d. Figure 5e shows both the voltage and current against sample type from lowest (Silk/PET) to highest output (EsNylon-Silk20/PVDF-PET) including the drop off for EsNylon-Silk30/PVDF-PET. In addition, to ascertain the electrical performance between electrospun nylon 66 and electrospun PVDF, nanofibers of nylon 66 and PVDF were deposited directly on the conductive aluminum foil. It was found that the TENG composed of electrospun nylon 66 and electrospun PVDF can generate an output voltage and a current of ∼13 V and −1 μA, respectively, as shown in Figure S2.

Further, transferred charges were calculated for all five types of t-TENGs to compare their electrical performances (Figure S3). The charge is calculated from the output current of the t-TENG by integration. As we know, ; therefore, , where  and  are the transferred charge and current, respectively. The transferred charge is maximum in the case of the EsNylon-Silk20/PVDF-PET-based t-TENG, that is, ∼0.04318 μC as compared to the other t-TENG devices (∼0.00224 μC for Silk/PET, ∼0.00623 μC for Silk/PVDF-PET, ∼0.02268 μC for EsNylon-Silk10/PVDF-PET, and ∼0.02434 μC for EsNylon-Silk30/PVDF-PET).

**Figure 6:**
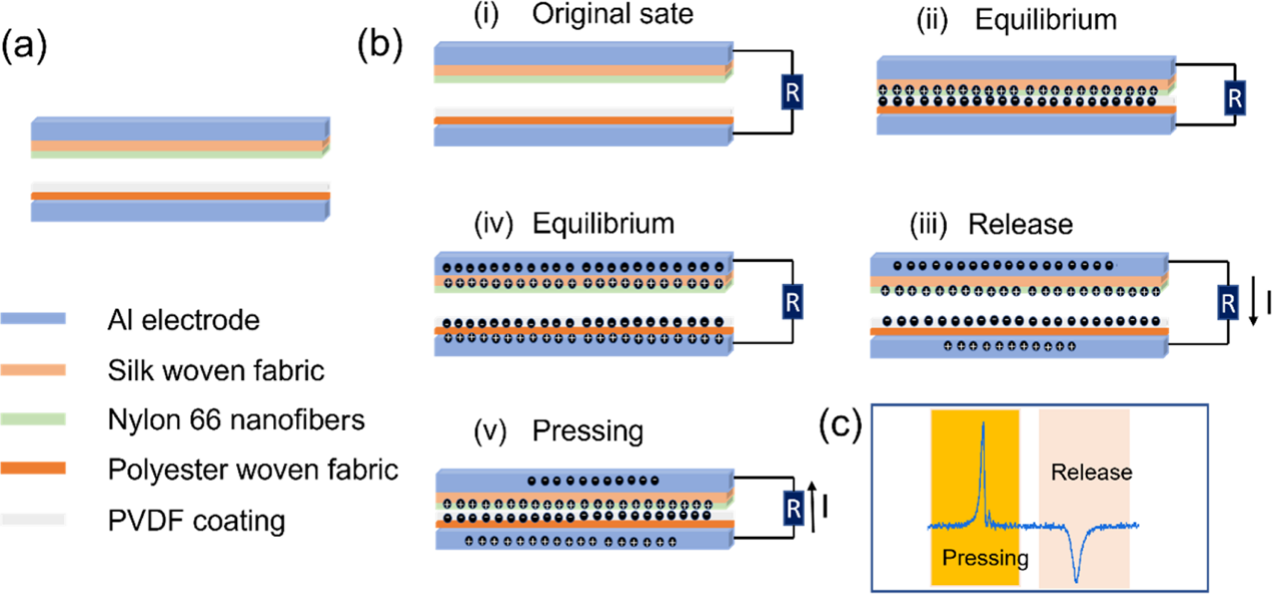
(a): Schematic of the EsNylon-Silk/PVDF-PET triboelectric nanogenerator composition; (b) working mechanism of the t-TENG; and (c) typical t-TENG signal due to pressing and releasing.

It is worth focusing briefly specifically on how the developed t-TENG (EsNylon-Silk/PVDF-PET) works in operation. This is briefly shown in Figure 6. Figure 6a shows a schematic of the device construction. Essentially, the tribo-positive contact layer is the nylon 66 nanofibers (on silk fabric) and the tribo-negative counter-surface is the PVDF coating (on PET fabric). Figure 6b shows a representative cycle of device operation. Initially, the surfaces are separated and electrically neutral (Figure 6b(i)). When the surfaces come into contact, electrons transfer from the tribo-positive nylon nanofibers to the tribo-negative PVDF (Figure 6b(ii)), resulting in a positive charge on the nylon surface and a negative charge on the PVDF surface. When the surfaces are moved apart, a potential difference between the electrodes is induced and opposite transferred charges develop on the electrodes due to electrostatic induction. Therefore, charge will flow from the bottom electrode to the top electrode through an external load to balance the potential difference (Figure 6b(iii)) until equilibrium is reached (Figure 6b(iv)). When the surfaces are then moved toward each other again, tribo-charge-induced potential difference will begin to reduce to zero so that the transferred charges now flow in reverse from the top electrode to the bottom electrode (Figure 6b(v)). Periodic repetition of the cycle causes electrons to flow back and forth between the two electrodes and generate an alternating voltage in the external circuit (Figure 6c). Here, one should remember that the silk and PET woven fabric may also be able to contribute to the induced surface charge on the interface surfaces even if coatings become worn off (because silk fabric is a good tribo-positive material, while PET is a good tribo-negative material).

**Figure 7:**
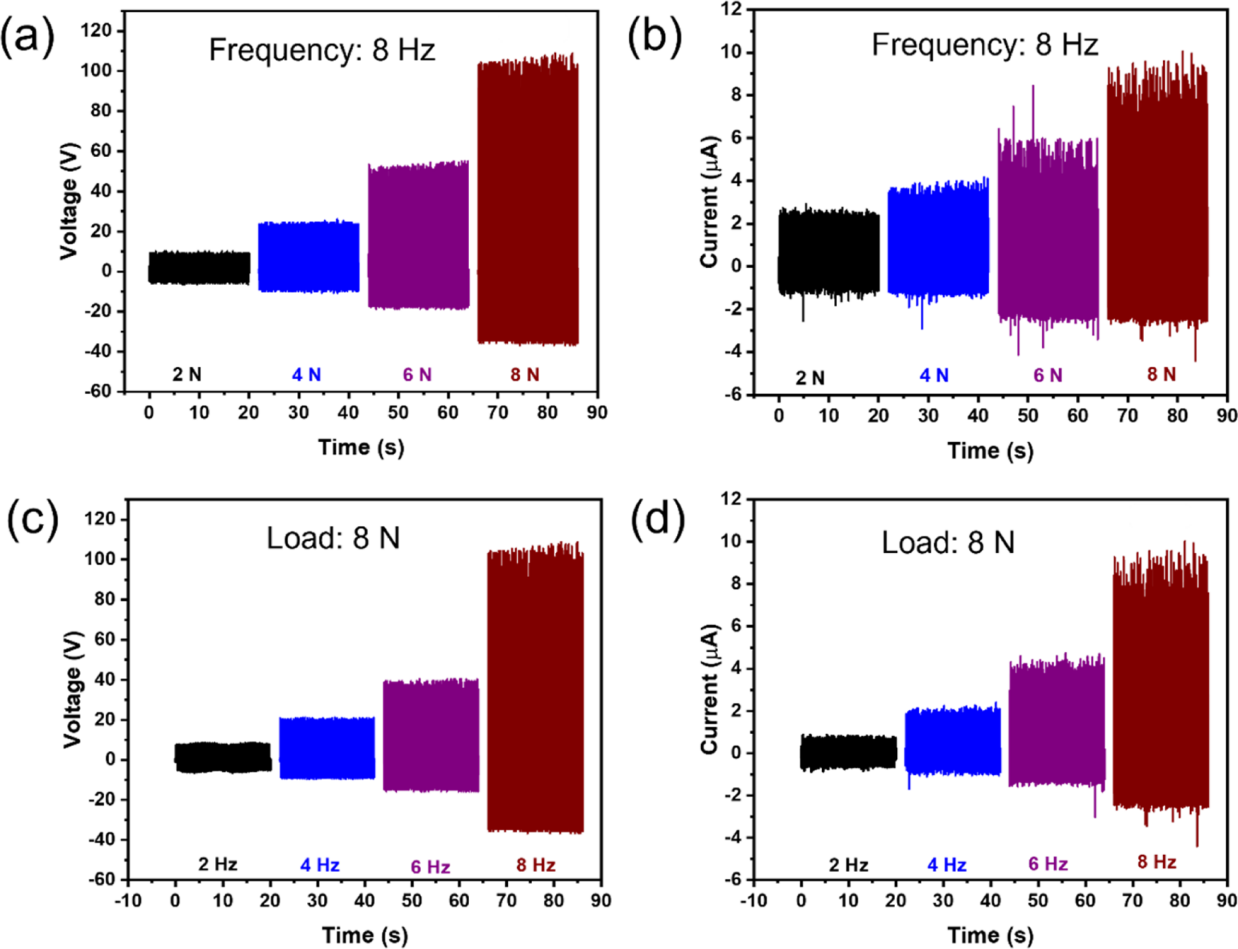
(a,b) Output voltage and current of the t-TENG vs contact force at a constant frequency of 8 Hz; (c,d) output voltage and current of the t-TENG against frequency with a constant contact force of 8 N (12.8 kPa). Note: result here applies to the optimum EsNylon-Silk20/PVDF-PET case.

Further characterization is now carried out for the t-TENG with optimum performance—that is, the EsNylon-Silk20/PVDF-PET case. Figure 7a,b shows that both the output voltage and current increase with applied contact force (frequency is constant here at 8 Hz). The output voltage and current increase from ∼10.33 V and 2.70 μA at 2 N to ∼105 V and ∼9.34 μA at 8N, respectively. Thus, with only a four times’ increase in contact force, the voltage and current have increased by 10.2 and 3.5 times, respectively. This confirms that the load-dependent TENG behavior noted for conventional non-fibrous TENGs in Min et al.^22^ is also very much applicable to the t-TENG here (as we would expect). As in,^22,60^ this is likely to be due to increasing contact area as the pressing force increases. In Figure 7c,d, contact force was held constant at 8 N (12.8 kPa pressure), and frequency was varied between 2 and 8 Hz. Similarly, here, both the output voltage and current increase with frequency (from ∼8.71 V and ∼0.87 μA at 2 Hz to ∼105 V and ∼9.34 μA at 8 Hz, respectively). This is fundamentally due to the increased rate of change of potential and capacitance between the electrodes brought about by the higher frequency (since ).^59,61^ The sensitivity to parameters such as contact force and frequency highlights the importance of taking account of these issues especially when comparing results from different labs.

**Figure 8:**
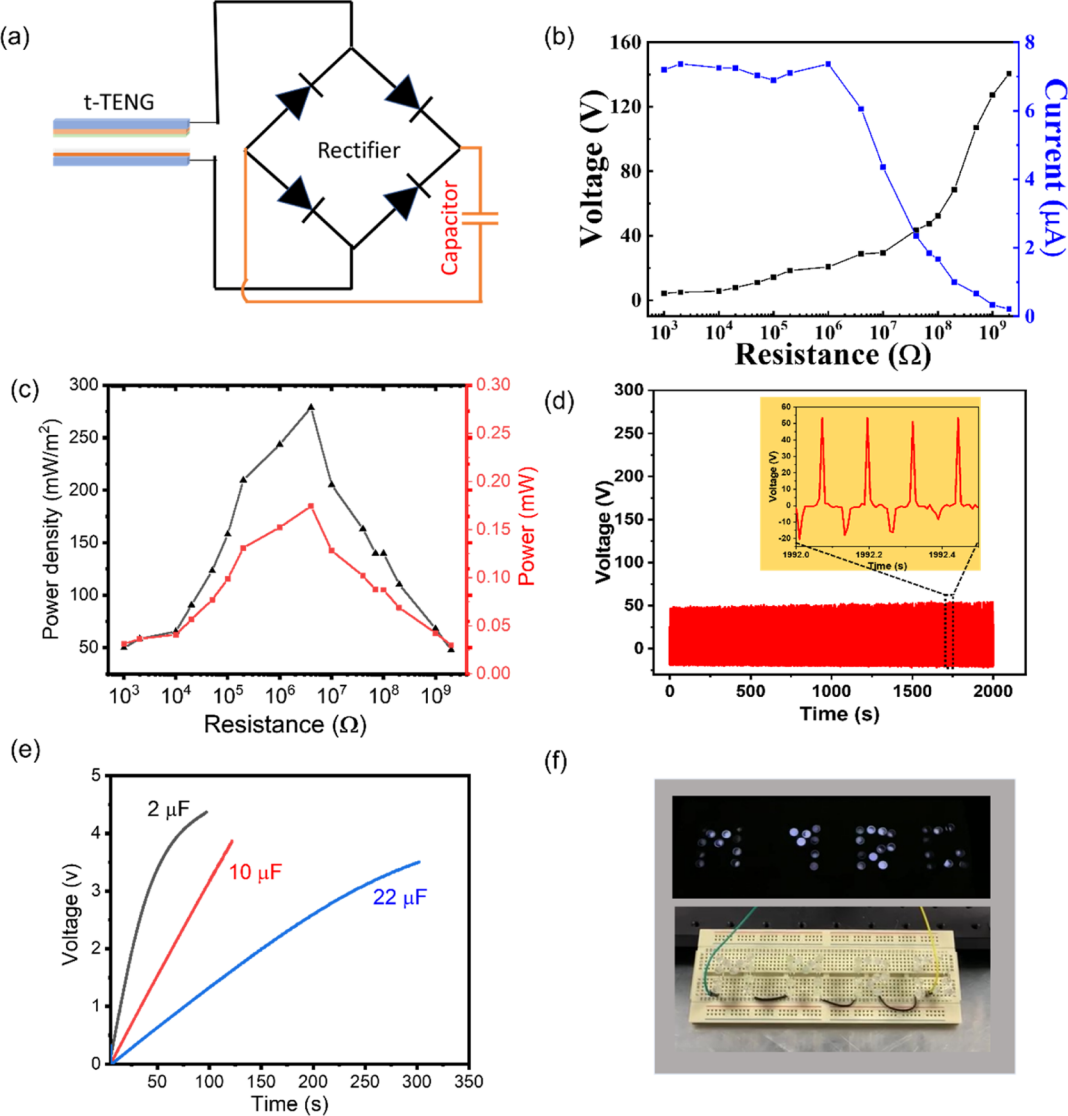
(a) Rectifier circuit diagram for charging of capacitors; (b) output voltage and current vs resistance; (c) power and power density vs resistance; (d) signal stability over time measured in terms of t-TENG output voltage under a 6 Hz frequency at a constant contact force of 8 N; (e) charging of 2, 10, and 22 μF capacitors using the developed t-TENG; and (f) 40 illuminated LEDs connected in series forming the word “MMRG”. Note: results here use the optimum EsNylon-Silk20/PVDF-PET case.

To determine the power characteristics of the device, the output voltage and current were measured over a range of electrical resistances (1 kΩ to 1 GΩ) at an 8 Hz frequency under a contact force of 8 N. The circuit shown in Figure 8a converts the AC TENG output to DC in order to charge the capacitors. The same circuit (as shown in Figure 8a) was also used to charge the capacitors having different capacities. Figure 8b shows the output voltage increasing with resistance and current decreasing as expected from Ohm’s law. The peak power and peak power density of the t-TENG were then determined as
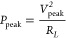
5
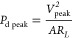
6where  and  are the peak output voltage and the load resistance, respectively, and  is the nominal t-TENG device area. Power and power density are shown in Figure 8c, and it can be seen that a peak power density of ∼280 mW/m^2^ is achieved at a resistance of about 4 MΩ. To assess the stability and durability of the fabricated t-TENG, the output voltage performance of the t-TENG device was measured with continuous loading for 2000 s (12,000 cycles) at a constant load and a frequency of 8 N and 6 Hz, respectively, as shown in Figure 8d. The result shows excellent stability and durability without any significant change in the output voltage. To investigate the self-powering capability, commercial capacitors having capacitances of 2, 10, and 22 μF were charged using the t-TENG device at a constant load of 8 N at an 8 Hz frequency (Figure 8e). The results show that the capacitors can be charged up to ∼4.38 V (for a 2 μF capacitor), ∼3.87 V (for a 10 μF capacitor), and ∼3.52 V (for a 22 μF capacitor) with charging rates of 0.045 V/s (2 μF capacitor), 0.031 V/s (10 μF capacitor), and 0.011 V/s (22 μF capacitor), respectively. Therefore, the electrical output from the developed t-TENG can be easily stored in a capacitor to supply power to portable and wearable electronic devices and sensors. In addition, to give a practical indication of t-TENG ability, 40 light-emitting diodes (LEDs) connected in series forming the acronym “MMRG” (Materials and Manufacturing Research Group) have been illuminated as shown in Figure 8f [with a corresponding video in the Supporting Information (Video S2)]. To demonstrate a promising wearable application of our developed TENG, the t-TENG device was attached on the metacarpophalangeal (MCP) joints of the human hand. Interestingly, it was found that the developed t-TENG can generate output voltages of ∼600 mV (for very slow movement), ∼2.6 V (for medium movement), and ∼12 V (for fast movement), respectively, as shown in Figure S4. An actual video of the finger-actuated t-TENG in operation is provided in the Supporting Information (Video S3). The voltage generated by the t-TENG due to the finger joint movement can illuminate a commercial LED as shown in the Supporting Information (Video S4). This experiment also demonstrates that the t-TENG has sufficient flexibility for wearable applications (Figure S5).

In Table 2, the max output from the present work is compared against papers from the literature where one of the tribo-contact surfaces is coated with a nanofibrous mat. Comparing results is difficult as devices in different works have different device areas and are usually tested under different contact pressures. Device area is easily accounted for by reporting power density *P*_d_ and short-circuit current density *J*sc. Here, we take the additional step of normalizing these by the average contact pressure *p* to account for different levels of mechanical loading. This is reasonable since the TENG output tends to be linearly proportional to (average) contact pressure at the low pressures typical in TENG tests (often <100 kPa). This produces a pressure-normalized power density (*P*_d_/*p*) in units of mW/N and a pressure-normalized current density (*J*_SC_/*p*) in units of mA/N (since the areas cancel, this is equivalent to power and current normalized by contact force as indicated by the units of power and current per unit force). Overall, despite the fact that the present TENG uses textile substrates (t-TENGs tend to have a considerably lower output), the results for power and current density are competitive with the other results which all have non-textile substrates. Of the eight studies we compare with in Table 2, the pressure-normalized max power density in the present work (*P*_d_^max^/*p*) is higher than four of the other results, and the pressure-normalized short-circuit current (*I*_SC_/*p*) is the third highest.

**Table 2:** Performance Comparison of the t-TENG Developed Here with Relevant TENG Devices Using Nanofiber Webs from the Literature

materials	pres. (*p*) (kPa)	*V*_O_ (V)	*J*_SC_ (mA/m^2^)	*P*_d_^max^ (mW/m^2^)	*P*_d_^max^/*p* (mW/N)	*J*_SC_/*p* (mA/N)	refs
PVDF-TrFE/MXene and nylon-11 nanofiber mat	23.3	180	140	4020	0.17	0.006	(29)
PVA/Lignin nanofibers and PTFE film	25	116	11.3	288	0.011	0.00045	(62)
PVDF/MoS_2_/CNT nanofibers and nylon fabric	13.9	300	3.19	134	0.0096	0.00023	(63)
TiO_2_/PAN/PTFE nanofibers and nylon film	5	60	0.15	48.6	0.009	0.00003	(64)
gelatin film and PLA nanofibers	31.25	500	10.6	5000	0.160	0.0003	(65)
mustard seeds and PVDF nanofibers	44.4	84	22	334	0.008	0.0005	(66)
Cu metal and PI nanofibers	37.5	95	188	2100	0.056	0.005	(67)
PVDF/ZnO NWs and nylon 11/ZnO NWs	100	330	10	3000	0.030	0.0001	(68)
**PA66 nanofiber-deposited silk fabric and PVDF polymer-coated PET fabric**	**12.8**	**101**	**24.5**	**280**	**0.0218**	**0.0019**	**present work**

The key advantage here is that the present modifications are made on cost-effective commercial silk and PET fabric substrates. This makes the approach in the present paper highly applicable for direct use in boosting TENG performance in wearable applications where flexibility and breathability are paramount factors.

## 4 Conclusions

This paper sets out an approach to developing a high-performance t-TENG using common commercial fabrics. Silk and PET were used as the base fabrics. Electrospun nylon 66 nanofibers were then deposited on silk fabric to form the tribo-positive layer and PVDF-coated PET fabric was used as the tribo-negative layer. FE-SEM and 3D optical profilometry were used to check the surface morphology and coating thickness, while the chemical signatures of the nylon and PVDF modifications were confirmed via XRD and FTIR analyses. The modifications produce a very significant boost in electrical output. The output voltage and short-circuit current density of the optimized coated device increased ∼17 times (from 5.85 to 100 V) and ∼16 times (from 1.6 to 24.5 mA/m^2^) when compared to the baseline Silk/PET combination, respectively. The maximum power density was 280 mW/m^2^ at a resistance of 4 MΩ. The performance boost is likely to result from a combination of two factors. First, the nylon 66 (and PVDF) clearly increases the tribo-positivity (and tribo-negativity) of the immediate contact layers and, second, the electrospun nylon nanofibers are likely to generate increased contact area at the interface. The effect of electrospinning deposition time and nanofiber deposition thickness on the TENG output was also explored and an optimum level established. The device showed excellent stability and durability over 12 000 cycles of testing—essentially, the nylon nanofibers and PVDF coating provide high output, while the silk and PET substrate fabrics confer strength and flexibility. The optimized device was shown to charge standard 2 and 22 μF capacitors to 4.4 V and 3.5 V in 1.5 to 5 min, respectively, and illuminate 40 LEDs. Operation of the device was successfully demonstrated on the MCP joints of the human hand. A key advantage is high output, but from a fully textile device with excellent flexibility. Another important benefit is construction based on commonly available and cost-effective textile fabrics (silk and PET). Thus, the high-performance t-TENG is an ideal candidate for use in powering wearable electronic devices and sensors. A similar approach can easily be adopted to boost output for a variety of textile combinations.

## Supporting Information Available

Output voltage video for the developed EsNylon-Silk20/PVDF-PET t-TENG (MOV)
LEDs illuminated using the developed EsNylon-Silk20/PVDF-PET t-TENG (MP4)
Output voltage for the t-TENG worn on the MCP finger joints of the human hand (MP4)
LEDs illuminated using the t-TENG actuated by movement of the MCP joints in the human hand (MP4)
Surface morphology (SEM scan) of the PVDF-coated PET woven fabric; electrical output from the TENG case having electrospun nylon 66 and electrospun PVDF deposited directly on the electrodes; transferred charge for each of the t-TENG instances; output voltage generated using a small-sized t-TENG (EsNylon-Silk20/PVDF-PET case) actuated via slow, medium, and fast movements of the MCP joints in the human hand; and a photograph showing the t-TENG attached at the MCP joints of the human hand and exhibiting the required flexibility for operation (PDF)

## Supplementary Material

am2c13092_si_001.mov
