# Supplementary material for: High-Performance Triboelectric Nanogenerators Based on Commercial Textiles: Electrospun Nylon 66 Nanofibers on Silk and PVDF on Polyester
Source: ACS Appl Mater Interfaces. 2022 Sep 23;14(39):44591–603. doi: 10.1021/acsami.2c13092 (PMC9542703; doi:10.1021/acsami.2c13092)
Supplement: Supplementary file 5 — am2c13092_si_005.pdf [file am2c13092_si_005.pdf]

Supporting Information

High performance triboelectric nanogenerator based on commercial textiles: electrospun nylon 66 nanofibres on silk and PVDF on polyester

Satyaranjan Bairagi[a*], Gaurav Khandelwal[b], Xenofon Karagiorgis[b], Shravan Gokhool[a], Charchit Kumar[a], Guanbo Min[b], and Daniel M. Mulvihill[a*]

[a] Materials and Manufacturing Research Group, James Watt School of Engineering, University of Glasgow, Glasgow, G12 8QQ, UK

[b] Bendable Electronics and Sensing Technologies (BEST) Group, James Watt School of Engineering, University of Glasgow, Glasgow, G12 8QQ, UK

*Corresponding authors: Daniel.Mulvihill@glashow.ac.uk (D.M. Mulvihill) and Satyaranjan.Bairagi@glasgow.ac.uk (S. Bairagi).

Fig. S1 shows a representative SEM image of the morphology of the PVDF coated PET woven fabric surface.

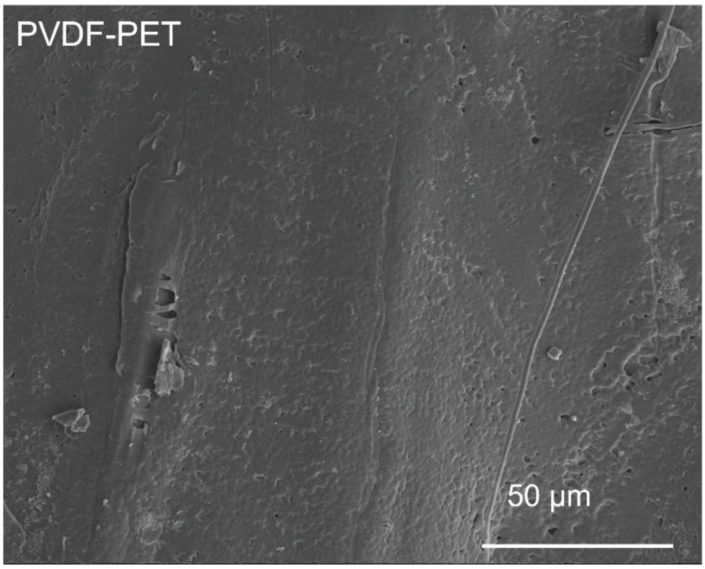

Fig. S1 Surface morphology (SEM scan) of the PVDF coated PET woven fabric.

Fig S2 indicates output voltage and current for TENG case having electrospun nylon 66 and electrospun PVDF deposited directly on the electrodes.

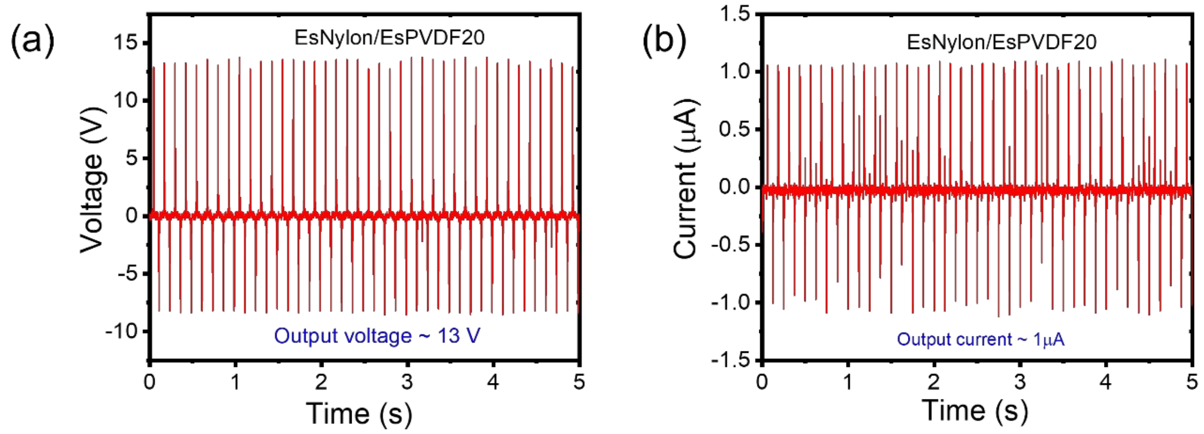

Fig. S2 Electrical output from the TENG case having electrospun nylon 66 and electrospun PVDF deposited directly on the electrodes: (a) output voltage and (b) output current.

Fig S3 indicates the transferred charge for the different t-TENG cases. Transferred change clearly increases as we move from the pristine Silk/PET case (0.002224 µC) to Silk/PVDF-PET (0.00623 µC) to EsNylon-Silk10/PVDF-PET (0.02268 µC) with max change transfer occurring for the EsNylon-Silk20/PVDF-PET case (0.04318 µC) and reducing for the EsNylon-Silk30/PVDF-PET case (0.02434 µC).

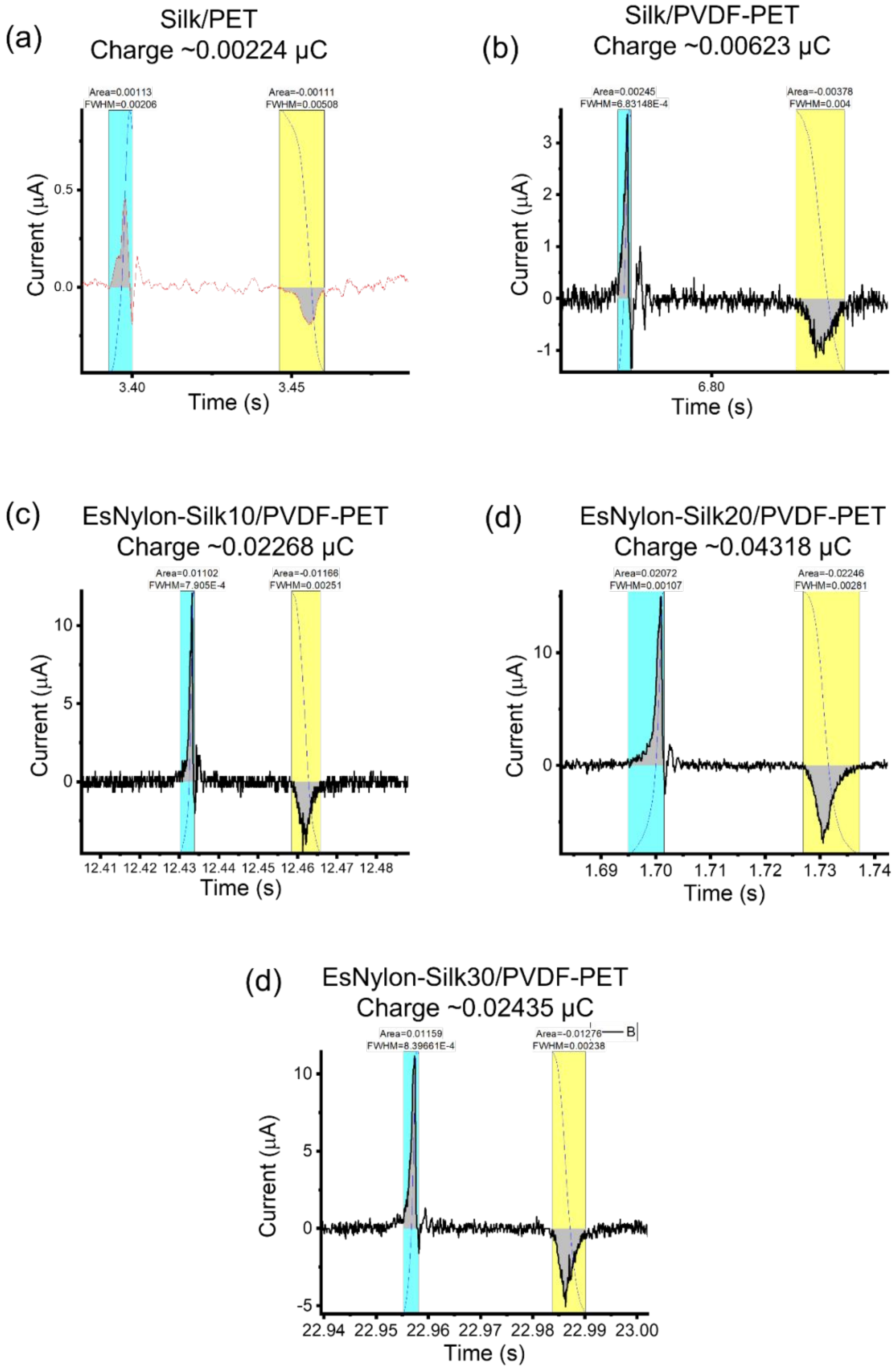

Fig. S3 Transferred charge for each of the t-TENG instances: (a) Silk/PET, (b) Silk/PVDF-PET, (c) EsNylon-Silk10/PVDF-PET, (d) EsNylon-Silk20/PVDF-PET, and (e) EsNylon-Silk30/PVDF-PET.

Fig S4 plots the output voltage for the wearable application of the t-TENG deployed on the metacarpophalangeal (MCP) joints of the human hand (using the optimised EsNylon-Silk20/PVDF-PET case).

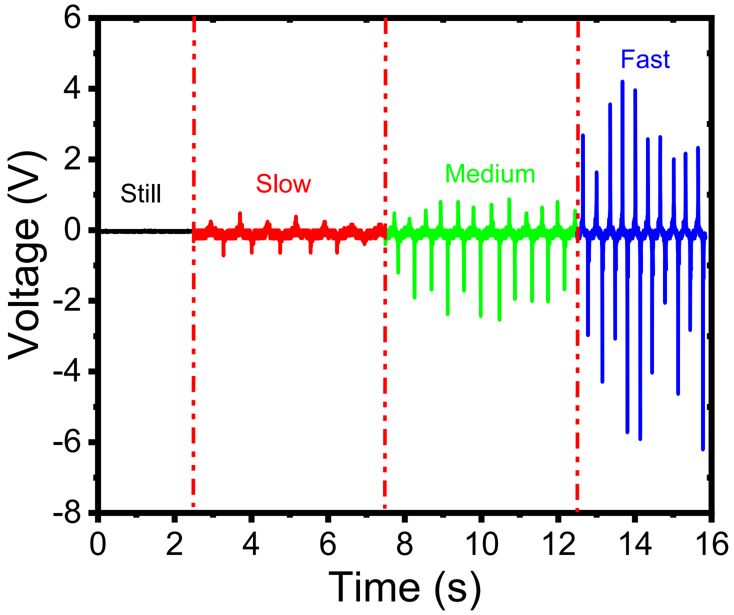

Fig. S4 Output voltage generated using a small size t-TENG (EsNylon-Silk20/PVDF-PET case) actuated via slow, medium, and fast movement of the metacarpophalangeal (MCP) joints in the human hand.

Fig S5 shows a photograph of the EsNylon-Silk20/PVDF-PET t-TENG attached to the metacarpophalangeal (MCP) joints of the human hand; thereby, demonstrating the flexibility of the developed device.

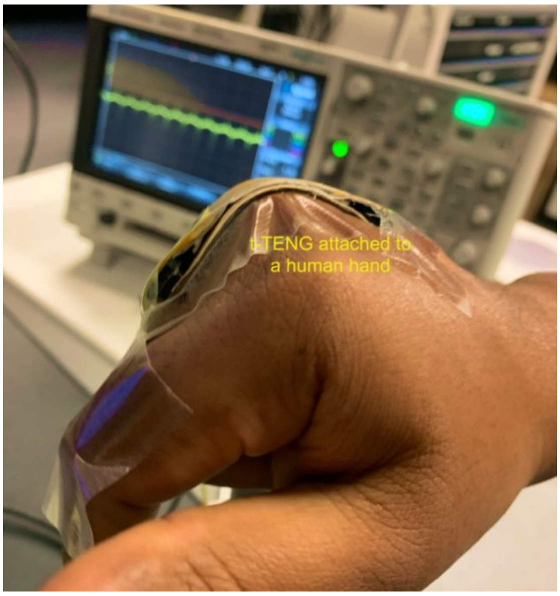

Fig. S5 Photograph showing the t-TENG attached at the metacarpophalangeal (MCP) joints of the human hand and exhibiting the required flexibility for operation.

Video S1: The output voltage video for the developed EsNylon-Silk20/PVDF-PET t-TENG (attached file)

Video S2: LEDs illuminated using the developed EsNylon-Silk20/PVDF-PET t-TENG (attached file)

Video S3: The output voltage for the t-TENG worn on the metacarpophalangeal (MCP) finger joints of the human hand (attached file)

Video S4: LEDs illuminated using the t-TENG actuated by movement of the metacarpophalangeal (MCP) joints in the human hand (attached file)
